# Detection of New Delhi metallo-beta-lactamase enzyme gene *bla*_NDM-1_ associated with the Int-1 gene in Gram-negative bacteria collected from the effluent treatment plant of a tuberculosis care hospital in Delhi, India

**DOI:** 10.1099/acmi.0.000125

**Published:** 2020-04-01

**Authors:** Amit Aggarwal, Manpreet Bhalla, Khan Hena Fatima

**Affiliations:** ^1^​ Department of Microbiology, National Institute of Tuberculosis and Respiratory Diseases, Delhi, India

**Keywords:** integron, New Delhi metallo-beta-lactamase, hospital sewage, effluent treatment plant, India

## Abstract

**Background:**

Organisms possessing the *bla*_NDM-1_ gene (responsible for carbapenem resistance) with a class-1 integron can acquire many other antibiotic resistance genes from the community sewage pool and become multidrug-resistant superbugs. In this regard, hospital sewage, which contains a large quantity of residual antibiotics, metals and disinfectants, is being recognized as a significant cause of antimicrobial resistance (AMR) origination and spread across the major centres of the world and is thus routinely investigated as a marker for tracing the origin of drug resistance. Therefore, in this study, an attempt has been made to identify and characterize the carbapenem-resistant microbes associated with integron genes amongst the organisms isolated from the effluent treatment plant (ETP) installed in a tertiary respiratory care hospital in Delhi, India.

**Methods:**

One hundred and thirty-eight organisms belonging to *Escherichia *, *Klebsiella *, *Pseudomonas* and *Acinetobacter* spp. were collected from the incoming and outgoing sewage lines of the ETP. Carbapenem sensitivity and characterization was performed by the imipenem and imipenem-EDTA disc diffusion method. Later DNA extraction and PCR steps were performed for the Int-1 and *bla*_NDM-1_ genes.

**Results:**

Of the 138 organisms, 86 (62.3 %) were imipenem-resistant (*P*<0.05). One hundred and twenty-four (89.9 %) organisms had one or both of the genes. Overall, the *bla*_NDM-1_ gene (genotypic resistance) was present in 71 % (98/138) of organisms. 53.6 % (74/138) organisms were double gene-positive (*bla*_NDM-1_ + Int-1), of which 40 were producing the metallo-beta-lactamase enzyme, making up almost 28.9 % (40/138) of the collected organisms.

**Conclusion:**

The current study strengthens the hypothesis that Carbapenem resistant organisms are in a high-circulation burden through the human gut and hospital ETPs are providing an environment for resistance origination and amplification.

## Introduction

Carbapenems (carbapenem, meropenem, imipenem) are currently our last option antimicrobial drugs in the battle against infections. Many times carbapenemase enzyme-producing Gram-negative bacteria are of superbug nature, wherein they are resistant to not only carbapenems but also to monobactums [1] and cephalosporins [2]. Many of the common nosocomial infectious agents, such as *Klebsiella pneumoniae *, *Pseudomonas aeruginosa* and *Acinetobacter baumannii,* are now presenting as superbugs and lead to complicated, difficult-to-treat infections of the urinary tract, blood, brain, lungs and wounds [3–7].

Structurally, either serine or zinc metal ion is present at the active sites of carbapenemase enzymes. Zinc-containing carbapenemase, like New Delhi metallo-β-lactamase (NDM), is also known as metallo-β-lactamase (MBL) or metallo-carbapenemase [8]. It was first isolated in an Indian-origin Swedish patient who travelled to India in 2008 [9] and has since then been identified in many countries, including Brazil, India and Southeast Asia [10–13]. There are many variants of NDM enzymes (e.g. NDM-1, 2, 3, 4 [14–16]) and the genes encoding them (*bla*_NDM-1, 2, 3_). Bacterial strains which are positive for *bla*_NDM_ (genotypic resistance marker gene) and also produce NDM enzymes are particularly dangerous because, (1) There is no routine standard phenotypic test for MBL detection; (2) Consequently there is a possibility of high prevalence of unrecognized asymptomatic carriers amongst general population; (3) There is lack of effective antibiotics against NDM-1-positive superbugs; and (4) Plasmids from these bacteria can undergo wide rearrangement and thus can cause widespread horizontal transmission of the antibiotic resistance gene (*bla*_NDM-1_) [17–20].

Many of the commensal bacterial strains become pathogenic and antibiotic-resistant after acquiring the *bla*_NDM-1_ gene. The association of this gene with DNA structures called gene cassettes and integrons is one of the possible explanations for its rampant spread [21]. Gene cassettes consist of DNA segments responsible for various functions such as antibiotic resistance, toxin–antitoxin, capsule, efflux systems, and many more. These are captured and expressed by other bacterial DNA segments (called integrons) that occur in bacteria from all environments and play an essential role in bacterial adaptation. There are many classes of integron [22], of which the classically studied class-1 integrons are the most mobile [23] and particularly widespread, occurring in anywhere from 10 to 50 % of commensal bacteria in humans, including in infants who have not yet been exposed to antibiotics [24–26]. Amongst the various cassette–integron systems identified, one for antibiotic resistance are the most studied. Class-1 integrons consist of specific genes such as *int-1* (which codes for the integron integrase enzyme), *Pc* (promotor), *qac* and *sul* at the 5′ and 3′ conserved regions. Various regions present in-between these two conserved regions have a site for the insertion of incoming antibiotic resistance gene cassettes. An integron may move between species and lineages either quickly or over evolutionary periods [27] and may acquire several gene cassettes placed one after another in a sequence analogous to songs on a music cassette tape. The association of cassettes with integrons helps in its recombination and expression according to the surrounding antibiotic, disinfectant or metal ion pressure. Moreover, the location of the integron–gene cassette system on plasmids makes their replication and horizontal transfer very easy [27]. It is to be emphasized that gene cassette expression is not entirely dependent on integrons; and expression may also occur through some non-integron-based promoter gene. Nevertheless, the insertion of a *bla*_NDM-1_-carrying gene cassette into a class-1 integron is a troublesome situation, as this integron can eventually acquire many other antibiotic resistance genes from the surroundings and convert a mono-drug-resistant bacterium into a multidrug-resistant superbug that is challenging to treat.

Human excreta have large number of susceptible and resistant bacteria, many of which contain integrons and resistance gene cassettes. The possibility of resistant bacteria being naturally selected over suseptible bacteria and transferring an integron-associated carbapenem resistance gene (via horizontal transfer) to non-pathogenic bacteria is much greater in the case of hospital sewage, where antimicrobial drugs are present in high amounts. Studies have shown that for any given antimicrobial, almost 30–90 % of the ingested drug is excreted unchanged from the gut [28]. Moreover, since these antibiotics and the resistance genes have long half-lives in the environment [29, 30], and are also difficult to remove during water treatment, they can eventually make their way into the food chain. Therefore sewage from health care institutes is now viewed as a significant cause of environmental contamination and as a marker for tracing the origin of resistance [31, 32]. Globally hospital sewage sampling is an attractive option over direct patient sampling for surveillance because it is representative of a large population and requires no consent with respect to ethical concerns [33–36]. The coexistence of integron and *bla*_NDM_ genes in the sewage and environment is worth attention and is relevant in the context of antimicrobial resistance emergence across the globe [37]. However, in the context of the Indian subcontinent, it is yet not clear how *bla*_NDM_ gene-positive strains producing MBL enzymes emerged and spread rampantly [38].

The National Institute of Tuberculosis and Respiratory diseases (NITRD) in Delhi, India is a tertiary respiratory care hospital with particular focus on tuberculosis treatment. Here the wastewater generated during bathing and washing activities is mixed with sewage water and pumped into the incoming line of a membrane-based ETP. Some amount of treated sewage water goes into horticulture activities within the campus, and the remaining is pumped away into the main outgoing municipality sewage line of the city. Patients at NITRD receive every kind of antimicrobial agent (antibacterial, antifungal, antitubercular and antiviral drugs) during their hospital stay. Here, at a rough estimate, around 32 % of the organisms isolated from the lower respiratory tract samples are carbapenem-resistant phenotypically. Thus, this scenario presents an exciting opportunity to study carbapenem resistance characteristics in an exclusive cohort of a tuberculosis care hospital. Therefore, a study was conceptualized in an attempt to identify integron-1-associated carbapenem resistance gene (*bla*_NDM-1_ gene) in four distinct Gram-negative bacteria (* Escherichia *, *Klebsiella *, *Pseudomonas* and *Acinetobacter *), belonging to the incoming and outgoing streams of the ETP. These four organisms are not only abundantly present in the human gut, but are also known agents of nosocomial infections.

## Methods

### Sample collection and organism identification

The study was conducted in the Department of Microbiology, NITRD, Delhi over 1 year from March 2018 to February 2019 wherein two sample collection days were randomly selected from each month. In this way, the total number of randomly selected days was 24. On any sample collection day, 100 ml sewage water samples were collected from the middle of the incoming and outgoing streams of the ETP after it had worked to full capacity for at least 30 min. Collected wastewater samples were streaked on 5 % sheep blood agar and MacConkey agar. The colonies of four common nosocomial infective agents, *Escherichia *, *Klebsiella *, *Pseudomonas* and *Acinetobacter *, were then identified by Gram staining and biochemical reactions based on standard protocols [39]. Organisms were later stocked in 20% glycerol broth solution [40]. Simultaneously, cultures of freshwater routinely supplied in hospital for drinking, washing and bathing purposes were also put up as controls to look for any residual presence of these organisms.

### Phenotypic detection of carbapenemase (serine carbapenemase/MBL)

**Fig. 1.**
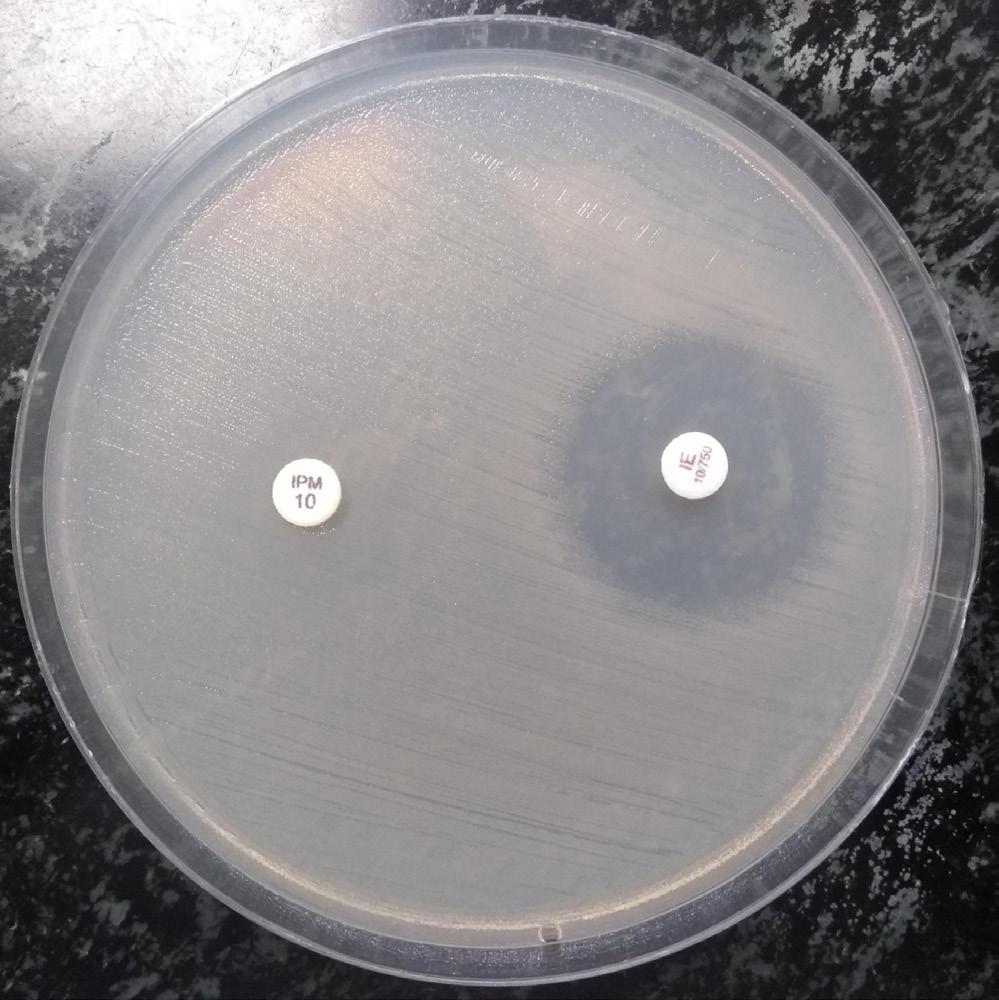
Enhancement of zone of inhibition by >7 mm with imipenem-EDTA antibiotic disc compared to imipenem disc alone (phenotypically positive for MBL enzyme).

All the organisms were tested against imipenem (10 µg) and an imipenem-EDTA (10/750 µg) drug combination using the disc diffusion method. With the imipenem disc, organisms with zone diameters of ≥23 mm, 20–22 mm and ≤19 mm were labelled as sensitive, intermediate and resistant, respectively. Then, the zone diameters of the above-identified resistant organisms were compared with the diameters of the combined imipenem- EDTA disc. Zone differences of >7 mm and <7 mm were taken to be indicative of the presence of presumptive metallo β lactamase (MBL) enzyme and serine β-lactamase (SBL) enzymes, respectively (Fig. 1) [41]. Due to resource constraints, these organisms were not tested for susceptibility against other routine antimicrobial drugs.

### Identification of integron-1 gene and *bla*_NDM-1_gene

This was done for all the collected organisms. The DNA extraction was performed according to methods described in the literature [42]. Briefly, two bacterial colonies were picked up from overnight growth on blood agar plates. These were placed in a test tube containing 25 µl of autoclaved double distilled water and heated at 95 °C for 10 min in a water bath. Tubes were then centrifuged for 10 min at 10000 r.p.m. The supernatant was collected and used for PCR. Uniplex touch-down PCR was set up with 25 µl of master mix solution containing 15 µl of nuclease-free water, 2.5 µl of 10× PCR buffer, 0.5 µl of 25 mM MgCl_2_, 0.5 µl of template DNA, 2 µl of 10 mM dNTP, 2 µl 10 µM forward primer, 2 µl of 10 µM reverse primer and 0.5 µl of 5 U/µl *Taq* DNA polymerase. DNA amplicons obtained were run on 1 % agar gel. PCR was set up using primers for integron-1 gene (FP- 5′-CCTCCCGCACGATGATC-3′; RP- 5′- TCCACGCATCGTCAGGC-3′) with a touch-down range from 58 to 54 °C and for *bla*_NDM-1_ gene (FP- 5′-GGTTTGGCGATCTGGTTTTC-3′; RP- 5′-CGGAATGGCTCATCACGATC-3′) with a touch-down range from 54 to 48 °C. The expected amplicon sizes were 280 and 621 bp for integron-1 and *bla*_NDM-1_ genes, respectively [43]. For optimization of PCR, ribonuclease-free water was included as a negative control. Non-specific band amplicons were expected during PCR as the collected samples were environmental in nature, but were ignored because no high-end sequencing techniques were used

Statistical analysis was performed using SPSS version 20 (IBM, New York, NY, USA) and chi-square test was performed. A *P*-value of <0.05 was considered to be indicative of a statistically significant difference.

## Results

Forty-eight sewage samples were gathered over the entire study period, 24 each from the incoming and outgoing sewage lines of the EFP. From these, 138 organisms belonging to *Escherichia* spp., *Klebsiella* spp., *Pseudomonas* spp. and *Acinetobacter* spp. were identified; 78 were from incoming line and 60 from outgoing line (*P*<0.05) (Table 1). None of the control plates inoculated with freshwater grew any of the above four genera of organisms.

**Table 1.** Distribution of organisms collected from incoming and outgoing sewage lines of the ETP

	Incoming ETP line	Outgoing ETP line	Total
* Escherichia* spp.	18	9	**27**
* Klebsiella* spp.	24	16	**40**
* Pseudomonas* spp.	24	24	**48**
* Acinetobacter* spp.	12	11	**23**
Total	**78**	**60**	**138**

Of the above 138 organisms, 62.3% (86/138) were phenotypically carbapenem-resistant, with 42 belonging to the incoming stream and 44 belonging to the outgoing stream. Only 37.7 % (52/138) of organisms were found to be carbapenem-sensitive (*P*<0.05) (Table 2). In the incoming wastewater stream, the numbers of resistant organisms in decreasing order were: *Klebsiella* spp., 23.1% (18/78); *Pseudomonas* spp., 17.9% (14/78); *Escherichia* spp., 10.3% (8/78); and *Acinetobacter* spp., 2.6% (2/78). By comparison, the numbers of resistant organisms in the outgoing stream were: *Pseudomonas* spp., 35% (21/60); *Klebsiella* spp., 16.6% (10/60); *Escherichia* spp., 11.7% (7/60); and *Acinetobacter* spp., 10% (6/60). Based on zone diameter differences between imipenem and combined imipenem-EDTA discs, 46.4% (64/138) of organisms were labelled as presumptive MBL enzyme-positive, and 15.9% (22/138) as presumptive serine**-**β-lactamase (SBL enzyme-positive). While the percentages of MBLs and SBLs in the incoming stream were 42.3% (33/78) and 11.5% (9/78), respectively, these increased to 51.7% (31/60) and 21.7 % (13/60) in the outgoing stream. Overall, there was a significant association between the presence of the resistance phenotype and increase in the zone diameter difference of >7 mm (*P*<0.05).

**Table 2.** Distribution of organisms, according to carbapenem (imipenem/imipenem-EDTA) sensitivity patterns and zone diameter difference

		Only Int-1 gene				Only *bla*_NDM-1_ gene				Int-1+*bla*_NDM-1_ genes				No gene detected				Total
Sensitivity; zone dia. diff.		Es	Kl	Ps	Ac	Es	Kl	Ps	Ac	Es	Kl	Ps	Ac	Es	Kl	Ps	Ac	
**S; <7** mm	**In**	3	1	1	2	2	1	1	1	2	3	2	1	2	0	1	1	**24**
	**Out**	0	1	0	1	0	0	0	1	1	1	2	1	0	0	0	0	**8**
**S; >7** mm	**In**	0	0	1	2	1	0	1	1	0	1	2	1	0	0	1	1	**12**
	**Out**	0	1	0	0	0	1	0	1	1	2	1	1	0	0	0	0	**8**
**R; <7 mm (SBLs)**	**In**	1	0	1	0	1	0	1	0	1	1	2	1	0	0	0	0	**9**
	**Out**	0	0	1	0	0	1	1	1	1	1	4	1	1	0	1	0	**13**
**R; >7 mm (MBLs)**	**In**	1	2	2	0	1	2	1	0	2	12	6	1	1	1	1	0	**33**
	**Out**	1	1	2	1	1	1	1	1	2	6	10	1	1	0	1	1	**31**
**TOTAL**		**6**	**6**	**8**	**6**	**6**	**6**	**6**	**6**	**10**	**27**	**29**	**8**	**5**	**1**	**5**	**3**	**138**
		**26**				**24**				**74**				**14**				

Ac, *Acinetobacter* spp; Es, *Escherichia* spp; In, incoming untreated wastewater stream; Kl, *Klebsiella* spp; MBLs, presumptive metallo beta-lactamase enzyme positive; Out, outgoing treated wastewater stream; Ps, *Pseudomonas* spp; R, imipenem resistant; S, imipenem sensitive; SBLs, presumptive serine beta-lactamase enzyme positive.

When these 138 organisms were tested for the presence of Int-1 and *bla*_NDM-1_ genes, 89.9% (124/138) of organisms had either or both of them (Table 2), while 10.1% (14/138) of organisms had none of them. 18.4% (26/138) had only Int-1 gene, 17.4% (24/138) had only *bla*_NDM-1_ gene, and 53.6% (74/138) strains were double-gene-positive. Of the 98 (71% of 138) *bla*_NDM-1_ positive genotypically resistant organisms, 52 (37.7% of 138) organisms came from the incoming untreated stream [66.7% (52/78)] and 46 (33.8% of 138) from the outgoing treated stream [76.7% (46/60)]. All the organisms contributed to this 10% increase of genotypically resistant organisms in outgoing stream, except *Escherichia* spp. Of the 74 (53.6 % of 138) double-gene-positive organisms (i.e. *bla*_NDM-1_ gene associated with Int-1 gene), 38 organisms (27.5% of 138) belonged to the incoming stream [48.7% (38/78)] and 36 (26.1% of 138) to the outgoing stream [60% (36/60)].

On comparing phenotypic sensitivities with genotypic resistance patterns, 65 (47.1% of 138) organisms were phenotypically imipenem resistant and also had *bla*_NDM-1_ gene. Of these 65 organisms, 13 (9.4% of 138) had only *bla*_NDM-1_ gene (of which 6 were from the incoming stream and 7 from the outgoing stream). The remaining 52 (37.7% of 138) organisms had both the genes (Int-1 with *bla*_NDM-1_) of which 26 belonged to the incoming stream and the remaining 26 to the outgoing stream. 21 organisms (15.2% of 138) showed discordance by being phenotypically resistant but *bla*_NDM-1_ gene-negative. Amongst the 52 (37.7% out of 138) phenotypically sensitive organisms, 19 *were bla*_NDM-1_ gene-negative but 33 *were bla*_NDM-1_ gene-positive. Overall, phenotypic/genotypic result concordance was seen in 60.8% (84/138) of organisms, wherein organisms were either imipenem-sensitive without the *bla*_NDM-1_ gene or were imipenem-resistant with the *bla*_NDM-1_ gene.

## Discussion

Injudicious use of antimicrobial agents has made antimicrobial resistance (AMR) among commensal gut microbes a significant problem. Hospital effluent treatment plants (ETPs) act as resistance amplifiers by not only naturally selecting resistant microbes in the presence of a large quantity of residual antimicrobials drugs, but also by spreading resistance mechanisms to non-pathogenic naïve organisms by horizontal gene transfer. Such microbes make their way through the food chain into the human gut, further amplifying the problem of AMR among commensal gut microbes.

The magnitude of this problem can be inferred from the results of the current study, where a large number of the organisms [62.3% (86/138)] collected from an ETP were found to be phenotypically resistant to a carbapenem drug (imipenem), with resistant organisms being found in substantial numbers in both the incoming [48.8% (42/86)] and outgoing [51.2% (44/86)] wastewater streams. Moreover, the spread of resistance from around half the incoming stream organisms [53.8% (42/78)] to around three-quarters of the outgoing stream organisms [73.3% (44/60)] further emphasizes the point that resistance amplification does occur in sewage through some kind of gene transfer; possibly horizontal gene transfer (*P*<0.05). In a similar survey of effluents from several geographically dispersed wastewater plants in the USA, 20.2% of the isolates were imipenem-resistant [44]. Studies from developing countries such as China and Brazil have reported 30–60% prevalence of carbapenem-resistant organisms [45–47].

In the current study, large numbers of bacteria were genotypically resistant by being *bla*_NDM-1_ gene-positive [71% (98/138)]. The figure was 66.7% (52/78) in the incoming stream but increased to a massive 76.7% (46/60) in the outgoing stream. Further, in many of these bacteria, this gene was found to be associated with the integron-1 gene [75.5% (74/98)], a combination which significantly increases the possibility of bacteria being either already a multidrug-resistant superbug or becoming one shortly. An 11% increase in the prevalence of such double-gene-positive organisms (*bla*_NDM-1_+Int-1 gene) while moving from the incoming stream [48.7% (38/78)] to the outgoing stream 60% (36/60) (*P*>0.05) shows that there is an ongoing process of active horizontal gene transfer, albeit not a significant one. In a similar study, led by Rice University, scientists found significant levels of NDM-1 enzyme and *bla*_NDM-1_ gene in the effluent released to the environment and even higher levels in dewatered sludge applied to soils [48]. Some studies have shown that the majority of the resistant microbes found in clinical sewage settings grow and persist in association with surface biofilms found on membranes and pipes of ETPs and not in the free planktonic state [49]. An 11% increase in the prevalence of double-gene-positive organisms in the current study could have happened because the ETP plant that NITRD hospital maintains is double membrane-based, which provided more surfaces for biofilm formation. However, the question of whether this frequency of gene transfer is amplified or reduced during much larger environmental mixing beyond ETP dilution is still being researched.

More than one-third of the organisms [37.7% (52/138)] isolated in this study showed full concordance in phenotypic/genotypic results by being carbapenem-resistant and positive for the *bla*_NDM-1_ gene associated with the integron-1 gene. These findings make apparent the disturbingly widespread presence of superbug-nature organisms amongst the pool of hospital sewage bacteria. Both int-1 and *bla*_NDM-1_ genes are not only essential for drug resistance, but their co-presence also gives an added advantage to the organism to evolve as a superbug. Although experiments to check the ability of such organisms to pick up other drug resistance genes from the sewage pool could not be performed in the current study due to lack of resources and technical expertise, studies have noted an assumption that the presence of antibiotics in wastewaters favours the proliferation of superbug bacteria [50–54]. Experiments have even shown that resistant bacterial strains have a selective survival advantage over susceptible strains even at sub-MIC levels, and such low concentrations can be found in wastewaters due to the dilution effect [55–59]. Amongst these phenotypically resistant multigene-positive organisms, around three-quarters [77.9% (40/52)] were presumptively metallo-beta-lactamase-positive and the remainder [23% (12/52)] were presumptively serine-beta-lactamase enzyme-positive. Such a high percentage of genotypically confirmed metallo-beta-lactamase-producing bacteria is a matter of concern, creating an endemic scenario leading to the level of an outbreak.

The current study has increased importance as the city of Delhi where it was performed has been projected to be a focus of superbug origination, and such surveillance studies can support the war against antimicrobial resistance by helping surveillance policy development. The present study is more of a prevalence study wherein an attempt has been made to determine the burden of phenotypic and genotypic resistance in the sewage. Complementing data by showing the linear relationship between antibiotic use in the hospital and the enrichment of resistance genes would have meant collecting a large number of samples and utilizing many resources, and thus was not attempted. It is to be understood that often PCR from environmental samples is ambiguous and sometimes shows non-specific band amplification. Although the lack of resources and technological expertise did limit the use of high-end 16SrRNA gene PCR amplification and sequencing technologies, the basic microbiological techniques implemented do complement the data presented during phenotypic and resistance gene identification. To the best of our knowledge, this is the first study from a selected urban tuberculosis/respiratory specialist hospital of a developing country, and provides an insight into the burden of carbapenem-resistant organisms carrying integron-1-associated metallo-beta-lactamases genes. Although there is currently no known correlation between the resistance mechanisms of anti-tubercular drugs and other common anti-bacterial drugs, the question of whether the resistance-determining regions for these two drug groups have any commonalities may become a topic of further investigation. The current study strengthens the hypothesis that superbugs are in a high-circulation burden through the human gut and hospital ETPs are providing the environment for resistance origination and amplification.

## References

[1] Cui X, Zhang H, Du H (2019). Carbapenemases in *Enterobacteriaceae*: detection and antimicrobial therapy. Front Microbiol.

[2] Schneider EK, Reyes-Ortega F, Velkov T, Li J (2017). Antibiotic-non-antibiotic combinations for combating extremely drug-resistant Gram-negative 'superbugs'. Essays Biochem.

[3] Yismaw G, Abay S, Asrat D, Yifru S, Kassu A (2010). Bacteriological profile and resistant pattern of clinical isolates from pediatric patients, Gondar university teaching Hospital, Gondar, Northwest Ethiopia. Ethiop Med J.

[4] Molla R, Tiruneh M, Abebe W, Moges F (2019). Bacterial profile and antimicrobial susceptibility patterns in chronic suppurative otitis media at the University of Gondar comprehensive specialized Hospital, Northwest Ethiopia. BMC Res Notes.

[5] Dagnew M, Yismaw G, Gizachew M, Gadisa A, Abebe T (2013). Bacterial profile and antimicrobial susceptibility pattern in septicemia suspected patients attending Gondar university Hospital, Northwest Ethiopia. BMC Res Notes.

[6] Getahun E, Gelaw B, Assefa A, Assefa Y, Amsalu A (2017). Bacterial pathogens associated with external ocular infections alongside eminent proportion of multidrug resistant isolates at the University of Gondar Hospital, Northwest Ethiopia. BMC Ophthalmol.

[7] Muluye D, Wondimeneh Y, Ferede G, Moges F, Nega T (2013). Bacterial isolates and drug susceptibility patterns of ear discharge from patients with ear infection at Gondar university Hospital, Northwest Ethiopia. BMC Ear Nose Throat Disord.

[8] Procop GW, Church DL, Hall GS, Janda WM, Koneman EW (2016). Koneman’s Color Atlas and Textbook of Diagnostic Microbiology.

[9] Yong D, Toleman MA, Giske CG, Cho HS, Sundman K (2009). Characterization of a new metallo-beta-lactamase gene, bla(NDM-1), and a novel erythromycin esterase gene carried on a unique genetic structure in Klebsiella pneumoniae sequence type 14 from India. Antimicrob Agents Chemother.

[10] Jovanović N, Jovanović J, Stefan-Mikić S, Kulauzov M, Aleksic-Dordević M (2008). [Mechanisms of bacterial resistance to antibiotics]. Med Pregl.

[11] Sambrook J, Fritsch EF, Maniatis T (1989). Molecular Cloning: a Laboratory Manual.

[12] Catana N, Herman V, Fodor I, Popa V (2009). Resistotypes in the APEC strains. L Sti Med Vet.

[13] Demerec M (1948). Origin of bacterial resistance to antibiotics. J Bacteriol.

[14] Yang H, Aitha M, Marts AR, Hetrick A, Bennett B (2014). Spectroscopic and mechanistic studies of heterodimetallic forms of metallo-β-lactamase NDM-1. J Am Chem Soc.

[15] Kaase M, Nordmann P, Wichelhaus TA, Gatermann SG, Bonnin RA (2011). NDM-2 carbapenemase in Acinetobacter baumannii from Egypt. J Antimicrob Chemother.

[16] Hornsey M, Phee L, Wareham DW (2011). A novel variant, NDM-5, of the new Delhi metallo-β-lactamase in a multidrug-resistant Escherichia coli ST648 isolate recovered from a patient in the United Kingdom. Antimicrob Agents Chemother.

[17] Akhtar J, Saleem S, Shahzad N, Waheed A, Jameel I (2018). Prevalence of metallo-β-lactamase IMP and VIM producing gram negative bacteria in different hospitals of Lahore, Pakistan. Pak J Zool.

[18] Ali K, Javaid U, Fatima K, Afreenish H, Maria O (2013). The frequency and antimicrobial sensitivity pattern of extended spectrum -lactamase (ESBLs) producing gram negative bacilli isolated from urine in a tertiary care hospital of Pakistan. Afr J Microbiol Res.

[19] Ain NU, Iftikhar A, Bukhari SS, Abrar S, Hussain S (2018). High frequency and molecular epidemiology of metallo-β-lactamase-producing gram-negative bacilli in a tertiary care hospital in Lahore, Pakistan. Antimicrob Resist Infect Control.

[20] Aneela S, Ul-Ain N-, Abrar S, Saeed M, Hussain S (2019). Distribution of extended-spectrum β-lactamase and metallo-β-lactamase-producing Pseudomonas aeruginosa in tertiary care hospitals of Lahore, Pakistan. JIMDC.

[21] Wright GD (2005). Bacterial resistance to antibiotics: enzymatic degradation and modification. Adv Drug Deliv Rev.

[22] Stokes HW, Hall RM (1989). A novel family of potentially mobile DNA elements encoding site-specific gene-integration functions: integrons. Mol Microbiol.

[23] Hall RM (2001). Integrons. Encycl Genet.

[24] Bailey JK, Pinyon JL, Anantham S, Hall RM (2010). Commensal Escherichia coli of healthy humans: a reservoir for antibiotic-resistance determinants. J Med Microbiol.

[25] Liu H, Wang H, Huang M, Mei Y, Gu B (2013). Analysis of antimicrobial resistance and class 1 integrons among strains from upper respiratory tract of healthy adults. J Thorac Dis.

[26] Tenaillon O, Skurnik D, Picard B, Denamur E (2010). The population genetics of commensal Escherichia coli. Nat Rev Microbiol.

[27] Gillings MR (2014). Integrons: past, present, and future. Microbiol Mol Biol Rev.

[28] Sarmah AK, Meyer MT, Boxall ABA (2006). A global perspective on the use, sales, exposure pathways, occurrence, fate and effects of veterinary antibiotics (vas) in the environment. Chemosphere.

[29] Zuccato E, Castiglioni S, Bagnati R, Melis M, Fanelli R (2010). Source, occurrence and fate of antibiotics in the Italian aquatic environment. J Hazard Mater.

[30] Le-Minh N, Khan SJ, Drewes JE, Stuetz RM (2010). Fate of antibiotics during municipal water recycling treatment processes. Water Res.

[31] Storteboom H, Arabi M, Davis JG, Crimi B, Pruden A (2010). Identification of antibiotic-resistance-gene molecular signatures suitable as tracers of pristine river, urban, and agricultural sources. Environ Sci Technol.

[32] Pruden A, Pei R, Storteboom H, Carlson KH (2006). Antibiotic resistance genes as emerging contaminants: studies in Northern Colorado. Environ Sci Technol.

[33] Fernández MDB, Torres C, Poma HR, Riviello-López G, Martínez LC (2012). Environmental surveillance of norovirus in Argentina revealed distinct viral diversity patterns, seasonality and spatio-temporal diffusion processes. Sci Total Environ.

[34] Madico G, Checkley W, Gilman RH, Bravo N, Cabrera L (1996). Active surveillance for Vibrio cholerae O1 and vibriophages in sewage water as a potential tool to predict cholera outbreaks. J Clin Microbiol.

[35] de Oliveira Pereira JS, da Silva LR, de Meireles Nunes A, de Souza Oliveira S, da Costa EV (2016). Environmental surveillance of polioviruses in Rio de Janeiro, Brazil, in support to the activities of global polio eradication initiative. Food Environ Virol.

[36] Hovi T, Shulman LM, van der Avoort H, Deshpande J, Roivainen M (2012). Role of environmental poliovirus surveillance in global polio eradication and beyond. Epidemiol Infect.

[37] Płusa T, Konieczny R, Baranowska A, Szymczak Z (2019). Narastająca oporność szczepów bakteryjnych Na działanie antybiotyków. The growing resistance of bacterial strains to antibiotics. Pol Merkur Lekarski.

[38] Lamba M, Graham DW, Ahammad SZ (2017). Hospital wastewater releases of Carbapenem-Resistance pathogens and genes in urban India. Environ Sci Technol.

[39] Pfaller Michael A, Louise LM, McAdam Alexander J (2019). Manual of Clinical Microbiology.

[40] De Paoli P (2005). Bio-banking in microbiology: from sample collection to epidemiology, diagnosis and research. FEMS Microbiol Rev.

[41] Tamma PD, Simner PJ (2018). Phenotypic detection of carbapenemase-producing organisms from clinical isolates. J Clin Microbiol.

[42] Pesce C, Kleiner VA, Tisa LS (2019). Simple colony PCR procedure for the filamentous actinobacteria Frankia. Antonie Van Leeuwenhoek.

[43] American Society of Microbiology (2016). Clinical Microbiology Procedures Handbook.

[44] Hoelle J, Johnson JR, Johnston BD, Kinkle B, Boczek L (2019). Survey of US wastewater for carbapenem-resistant Enterobacteriaceae. J Water Health.

[45] Chandran SP, Diwan V, Tamhankar AJ, Joseph BV, Rosales-Klintz S (2014). Detection of carbapenem resistance genes and cephalosporin, and quinolone resistance genes along with oqxAB gene in Escherichia coli in hospital wastewater: a matter of concern. J Appl Microbiol.

[46] Zhang C, Qiu S, Wang Y, Qi L, Hao R (2014). Higher isolation of NDM-1 producing Acinetobacter baumannii from the sewage of the hospitals in Beijing. PLoS One.

[47] Ferreira AE, Marchetti DP, De Oliveira LM, Gusatti CS, Fuentefria DB (2011). Presence of OXA-23-producing isolates of Acinetobacter baumannii in wastewater from hospitals in southern Brazil. Microb Drug Resist.

[48] Luo Y, Yang F, Mathieu J, Mao D, Wang Q (2014). Environmental Science & Technology Letters.

[49] Aminov RI (2011). Horizontal gene exchange in environmental microbiota. Front Microbiol.

[50] Li J, Cheng W, Xu L, Strong PJ, Chen H (2015). Antibiotic-Resistant genes and antibiotic-resistant bacteria in the effluent of urban residential areas, hospitals, and a municipal wastewater treatment plant system. Environ Sci Pollut Res Int.

[51] Rizzo L, Manaia C, Merlin C, Schwartz T, Dagot C (2013). Urban wastewater treatment plants as hotspots for antibiotic resistant bacteria and genes spread into the environment: a review. Science of The Total Environment.

[52] Bouki C, Venieri D, Diamadopoulos E (2013). Detection and fate of antibiotic resistant bacteria in wastewater treatment plants: a review. Ecotoxicol Environ Saf.

[53] Ng C, Tay M, Tan B, Le T-H, Haller L (2017). Characterization of metagenomes in urban aquatic compartments reveals high prevalence of clinically relevant antibiotic resistance genes in wastewaters. Front Microbiol.

[54] He P, Yu Z, Shao L, Zhou Y, Lü F (2019). Fate of antibiotics and antibiotic resistance genes in a full-scale restaurant food waste treatment plant: implications of the roles beyond heavy metals and mobile genetic elements. J Environ Sci.

[55] Munck C, Albertsen M, Telke A, Ellabaan M, Nielsen PH (2015). Limited dissemination of the wastewater treatment plant core resistome. Nat Commun.

[56] Andersson DI, Hughes D (2014). Microbiological effects of sublethal levels of antibiotics. Nat Rev Microbiol.

[57] Rizzo L, Manaia C, Merlin C, Schwartz T, Dagot C (2013). Urban wastewater treatment plants as hotspots for antibiotic resistant bacteria and genes spread into the environment: a review. Sci Total Environ.

[58] Bengtsson-Palme J, Larsson DGJ (2016). Concentrations of antibiotics predicted to select for resistant bacteria: proposed limits for environmental regulation. Environ Int.

[59] Gullberg E, Cao S, Berg OG, Ilbäck C, Sandegren L (2011). Selection of resistant bacteria at very low antibiotic concentrations. PLoS Pathog.

